# Novel technique for intra-operative evacuation of an obstructed bowel (ASA technique)

**DOI:** 10.1093/jscr/rjag644

**Published:** 2026-08-06

**Authors:** Abdullah Saad Alqahtani, Nasser Alsanea, Reem Awad Alharbi, Asim Abdullah Elyas, Saad Obaid Alghamdi, Toka M Hussein, Mahmoud R A Hussein

**Affiliations:** Department of Surgery, Armed Forces Hospitals Southern Region, Khamis Mushait, KSA, Kingdom of Saudi Arabia; Department of Surgery, College of Medicine, Princess Nourah Bint Abdulrahman University, Riyadh, KSA, Kingdom of Saudi Arabia; Department of Surgery, College of Medicine, Princess Nourah Bint Abdulrahman University, Riyadh, KSA, Kingdom of Saudi Arabia; Department of Surgery, Armed Forces Hospitals Southern Region, Khamis Mushait, KSA, Kingdom of Saudi Arabia; Department of Surgery, Armed Forces Hospitals Southern Region, Khamis Mushait, KSA, Kingdom of Saudi Arabia; Faculty of Medicine, Sohag University, Sohag, Egypt; Department of Pathology, Faculty of Medicine, Assiut University Hospitals, Assiut, Egypt

**Keywords:** ASA, bowel, evacuation, obstruction, technique, intra-operative

## Abstract

Intra-operative management of obstructed distended bowel poses a risk of spillage and contamination. Here we describe our novel surgical technique for intra-operative evacuation of a distended, stool-filled obstructed bowel using a standard Mayo’s stand cover. The bowel segment proximal and distal to the obstruction is mobilized. The bowel is transected distally. A Mayo's stand cover is attached to the edge of the incision with clamps, creating a sterile collection pouch. The mobilized bowel is exteriorized into the bag. An enterotomy is made just proximal to the obstruction, allowing the stool to evacuate into the bag. Manual antegrade bowel decompression is used to assist emptying. The bowel is then transected proximally and both the resected segment and the bag are removed. The procedure continues with primary anastomosis or stoma formation. Our novel technique provides an effective method to safely decompress a dilated bowel segment while minimizing contamination.

## Introduction

Surgical management of bowel obstruction, particularly in cases involving massively distended, stool-filled bowel, carries a risk of contamination and technical difficulty during closure. In many instances, decompression is necessary before performing a safe anastomosis or stoma. Several methods exist, but many are costly or complex [1–3]. The disposable plastic Mayo stand cover is a sterile, disposable, fluid-resistant plastic drape readily available in operating rooms worldwide [4, 5]. Here, we present the Abdullah Saad Alqahtani (ASA) technique, which utilizes this readily accessible item to achieve effective and clean bowel evacuation during surgery.

## Case series and surgical technique

The steps of our novel surgical technique are as follows. First, the bowel is mobilized both proximal and distal to the obstructing lesion. During this step, feeding and draining vessels are ligated in accordance with standard surgical principles. Once adequate mobilization is achieved, the bowel is transected immediately distal to the obstruction using a linear cutting stapler.

Next, a sterile Mayo’s stand cover (Manufacturer Arabian Medical Products Manufacturing Company (ENAYAH) Brand Name ENAYAH City Riyadh Country Kingdom of Saudi Arabia) is prepared by securing its open edge to the surgical incision with atraumatic clamps. The remaining portion of the cover is allowed to hang over the side of the operating table, forming a sterile collection pouch. Following this, the free end of the mobilized segment of bowel that contains the obstruction is exteriorized and placed into the Mayo stand cover pouch, allowing for contained handling of its contents.

A controlled enterotomy is then created just proximal to the obstructing lesion. This permits the evacuation of bowel contents directly into the sterile cover. Manual antegrade gentle decompression (‘milking’) is employed to facilitate thorough emptying of the bowel. Intra-operative bowel lavage may also be performed at the surgeon’s discretion.

**Figure 1:**
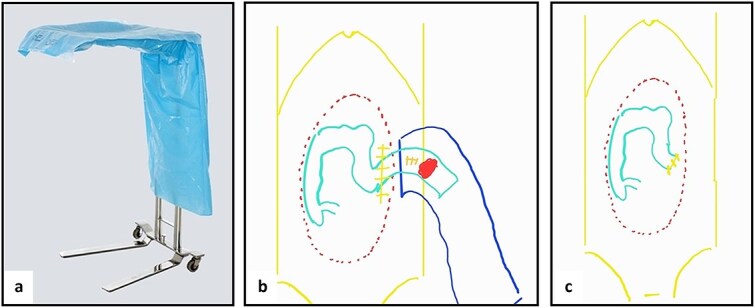
Diagrammatic representation of ASA technique. (a) Mayo stand cover attached to incision. (b) Exteriorized bowel placed in cover. (c) Enterotomy and evacuation process.

**Figure 2:**
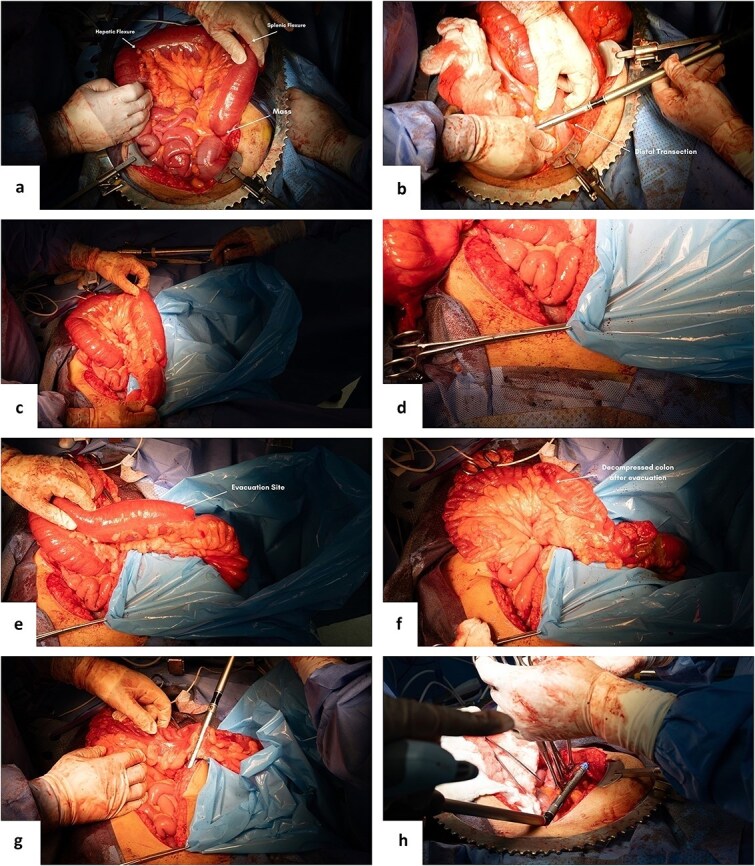
Steps of ASA technique for intra-operative evacuation of an obstructed bowel using a Mayo’s stand cover. This technique describes a contained method for decompressing an obstructed bowel before resection to minimize contamination. The key steps are as follows: (a) The bowel is freed and then stapled below the obstruction. (b) A sterile Mayo's stand cover is attached to the incision to form a hanging pouch. (c) An opening is made proximal to the obstructing lesion, allowing stool to be emptied ("milked") directly into the sterile cover. (d) After decompression and any needed washout, the bowel is transected above the lesion. The entire specimen and the pouch containing the stool are removed together. (e–f) The operation continues with either an anastomosis or stoma creation.

After complete evacuation and decompression of the bowel, additional mobilization may be carried out if needed. The bowel is subsequently transected proximal to the lesion, and both the resected bowel segment and the Mayo’s stand cover containing the stool contents are removed from the surgical field. At this point, the operation proceeds with the next planned step, which may include either primary anastomosis or stoma formation, depending on the clinical scenario. The technique was used in several cases thereafter. A summary of these steps are shown in Figs 1 and 2 and Video 1.

Ethical approval was obtained from the hospital’s institutional review board. A case series was conducted involving five patients who presented with intestinal obstruction and were managed intra-operatively using the ASA technique with a Mayo's stand cover. The cohort consisted of five patients with a median age of 57 years, ranging from 46 to 82 years. There was a notable male predominance, as the series included four male patients and one female patient. Clinically, all five patients presented with the classic triad of large bowel obstruction symptoms, which included abdominal pain, constipation, and abdominal distention.

Pre-operative radiological imaging confirmed the diagnosis of an obstructing colon tumour in every case, with associated proximal bowel dilatation. The distribution of the obstructing tumors varied among the patients, with two tumors located at the splenic flexure, two in the descending colon, and one in the sigmoid colon. Intra-operative findings correlated precisely with the radiological findings in all five cases, and each patient underwent surgical total mesocolonic resection of the tumor-bearing segment with primary anastomosis. The use of the ASA technique facilitated the management of the dilated proximal bowel during these procedures. Following surgery, the final histopathological examination of the resected specimens confirmed the diagnosis of adenocarcinoma in all five cases. The procedures were performed over an 8-month period from June 2025 to February 2026, demonstrating the recurring applicability of the technique in clinical practice. A summary of clinical presentations and outcomes of the five patients is shown in Table 1.

**Table 1:** ASA technique for intra-operative evacuation of an obstructed bowel using a Mayo’s stand cover in five patients with intestinal obstruction

Case No.	Age	Sex	Date	Clinical presentation	Radiological findings	Intra-operative findings	Procedure	Histological diagnosis
1	56	M	17 June 2025	Abdominal pain, constipation, and distension	Sigmoid obstructing tumor with proximal bowel dilatation	Sigmoid tumor with distended large and small bowel	Resection with primary anastomosis	Adenocarcinoma
2	57	M	14 July 2025	Abdominal pain, constipation, and distension	Distal transverse/splenic flexure obstructing tumor with proximal bowel dilatation	Tumor at splenic flexure with distended proximal bowel	Resection with primary anastomosis	Adenocarcinoma
3	82	F	24 July 2025	Abdominal pain, constipation, and distension	Distal transverse/splenic flexure obstructing tumor with proximal bowel dilatation	Tumor at splenic flexure with distended proximal bowel	Resection with primary anastomosis	Adenocarcinoma
4	46	M	14 January 2026	Abdominal pain, constipation, and distension	Left colon obstructing mass with proximal bowel dilatation	Tumor at descending colon with distended proximal bowel	Resection with primary anastomosis	Adenocarcinoma
5	70	M	15 February 2026	Abdominal pain, constipation, and distension	Left colon obstructing mass with proximal bowel dilatation	Tumor at descending colon with distended proximal bowel	Resection with primary anastomosis	Adenocarcinoma

## Discussion

Previous techniques—like needle aspiration of gas or sealed trocar suction—focus on intra-operative decompression while minimizing wound contamination [4, 5]. Our ASA technique extends this principle by utilizing a Mayo’s stand cover as a flexible, sterile collecting pouch that allows for full evacuation of bowel content without intra-abdominal spillage. Manual decompression has been shown, in our hands, to be safe; appears as safe as colonic irrigation based on published data and may lead to fewer anastomotic leaks and comparable postoperative outcomes [1]. Our data supports the rationale for using a controlled mechanical evacuation through the use of the ASA technique, which combines physical decompression without contamination of the surgical field.

The ASA technique provides a novel, simple, and reproducible, and resource-friendly approach to intra-operative bowel evacuation in cases of bowel obstruction. Moreover, utilizing surgical supply readily available in nearly all operating rooms is a form of cost-efficiency. The preservation of a sterile surgical field helps improve the outcome for a bowel anastomosis and prevents deep-seated postoperative abdominal infections. The ASA technique is simple In contrast to more complex or expensive bowel decompression methods [1, 5, 6].

Finally, the use of a Mayo’s stand cover offers a pragmatic alternative, particularly in low-resource settings or emergency situations. However, the success of this technique hinges on adequate mobilization of the obstructed bowel without compromising its blood supply. It is important to note that the ASA technique is not appropriate for cases where the proximal bowel is ischemic, friable, perforated or otherwise unhealthy.

To conclude, the ASA technique is a safe, effective, and practical method for intra-operative bowel evacuation in handling cases of bowel obstruction especially during the emergency setting. It helps prevent contamination, facilitates decompression, and prepares the surgical field for safe continuation of surgery. Its simplicity and cost-effectiveness make it particularly valuable in resource-limited environments.

## Supplementary Material

ASA_technique_rjag644
